# Antitumor activities of Quercetin and Green Tea in xenografts of human leukemia HL60 cells

**DOI:** 10.1038/s41598-018-21516-5

**Published:** 2018-02-22

**Authors:** Andrana Karla Calgarotto, Victor Maso, Gilberto Carlos Franchi Junior, Alexandre Eduardo Nowill, Paulo Latuf Filho, José Vassallo, Sara Teresinha Olalla Saad

**Affiliations:** 1 Hematology and Transfusion Medicine Center-University of Campinas/Hemocentro-UNICAMP, Instituto Nacional de Ciência e Tecnologia do Sangue, Campinas, São Paulo Brazil; 2 Onco-Hematological Child Research Center, Faculty of Medical Sciences, University of Campinas - UNICAMP, Campinas, São Paulo Brazil; 3 Department of Pathology, Faculty of Medical Sciences, Laboratory of Investigative and Molecular Pathology, CIPED, FCM-UNICAMP, Campinas, Brazil

## Abstract

Quercetin is one of the most abundant flavonoids, present in fruits and vegetables and has been shown to have multiple properties capable of reducing cell growth in cancer cells. Green tea is a widely consumed beverage, known for a potential source of free radical scavenging and anti-cancer activities. Herein, we investigate the *in vivo* antitumor efficacy of quercetin and green tea in human leukemia. Human tumors were xenografted into NOD/SCID mice. Quercetin and green tea reduced tumor growth in HL-60 xenografts accompanied by decreased expression of anti-apoptotic proteins, BCL-2, BCL-XL and MCL-1 and increased expression of BAX, a pro-apoptotic protein. Moreover, caspase-3 was activated to a greater extent after quercetin and green tea treatment. Quercetin and green tea also mediated G1 phase cell cycle arrest in HL-60 xenografts. Treatment with quercetin and green tea induced conversion of LC3-I to LC3-II as well as activation of autophagy proteins, suggesting that quercetin and green tea initiate the autophagic progression. We have provided evidence that quercetin and green tea induces signaling at the level of apoptosis, cell cycle and autophagy which converge to antigrowth effects in HL-60 xenograft mice suggesting that these compounds may be a compelling ally in cancer treatment.

## Introduction

Acute myeloid leukemia (AML) is a disorder of hematopoietic stem cells in which leukemic cells acquire mutations that confer high turnover capacity, altered hematopoietic differentiation, and increased proliferative capacity. AML therapy remains a challenge for scientists and hematologists worldwide, and represents an urgent medical need. There are several plant-derived compounds with established clinical uses for treating hematological malignancies. The vinca alkaloids, vincristine and vinblastine, the first plant-derived anticancer agents to be approved by the U.S. FDA, are used for the treatment of lymphomas, including Hodgkin’s disease, and for acute lymphoblastic leukemias in combination chemotherapy^1,2^. Furthermore, etoposide, an effective compound for different types of leukemias and lymphomas, and teniposide with activity against various types of hematological malignancies, either alone or in combination chemotherapy, are semi-synthetic plant derivatives^3,4^. Studies have identified natural compounds, quercetin and green tea, with real potential to reduce cell proliferation and induce neoplasic cell death^5,6^.

Quercetin is a polyphenol present in fruits and vegetables and has been reported as being useful for the treatment of metabolic and inflammatory disorders^7^. Quercetin is known to contribute as an apoptosis inductor decreasing the growth of tumors in brain, liver, colon, and other tissues and inhibiting the spread of malignant cells^5,7^.

Green tea is obtained from the leaves of the plant *Camellia sinensis*, which has been consumed as a drink for thousands of years in China and Japan and whose therapeutic properties have been observed over time by the population. Polyphenols are the main chemical constituents of *C*. *sinensis*. Epidemiological studies and murine models have identified promising results for green tea and its constituents in reducing the risk of cancer in different organs^8–11^. Green tea inhibited tumor growth in xenograft models by suppressing the metastasis on metastasis-specific mouse mammary carcinoma 4T1 cells^12^. In addition, epigallocatechin-3-gallate (EGCG), the major polyphenol of green tea, has been shown to inhibit proliferation and induce apoptosis through caspase-3 activation and to decrease VEGF expression in esophageal squamous cell carcinoma *in vitro* and *in vivo*^8,13^.

The present study shows through the inhibition of xenografts growth of HL-60 cells, that quercetin and green tea have an antileukemia ability. Our study further shows that the signaling pathways of apoptosis, cell cycle and autophagy are modulated in tumor-bearing mice describing the therapeutic efficacy of quercetin and green tea on human leukemia.

## Results

### Quercetin and Green tea reduce tumor growth in HL60 xenografts

**Figure 1:**
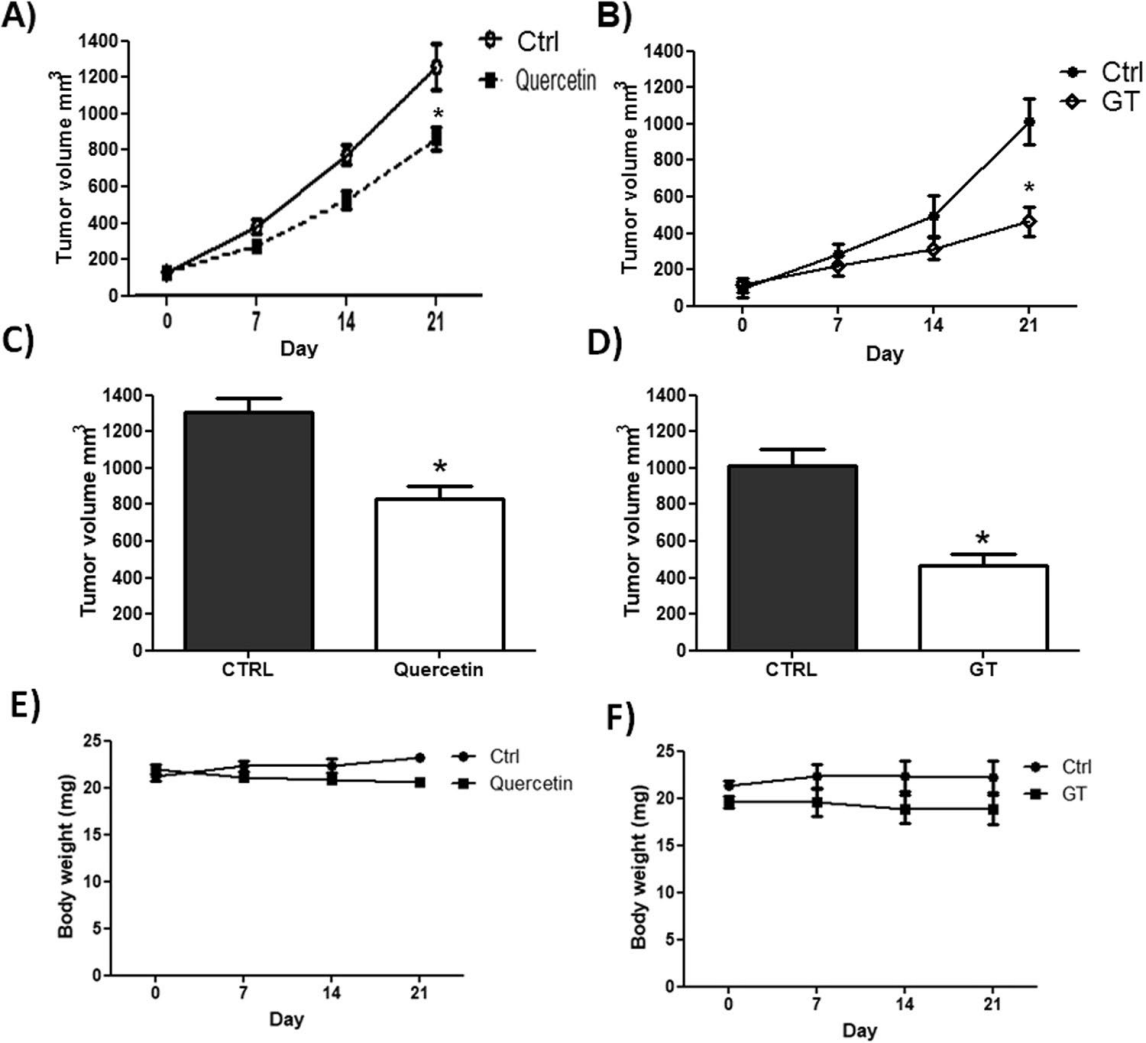
Quercetin (**A**) and (**B**) Green tea reduce tumor growth in HL60 xenografts. (**C**) Quercetin (**D**) and Green tea tumor volume after 21 days of treatment. The body weights of the xenograft mice treated with Quercetin (**E**) and Green tea (**F**). NOD/SCID mice with HL-60 tumors were treated or not with quercetin (120 mg/kg) once every 4 days or green tea (100 mg/kg) every day during 21 days. Every 7 days the tumor volumes were evaluated according to the following formula: tumor volume (mm^3^) ¼ (length × width2)/2. The control group received equal amounts of vehicle solution, as indicated in Materials and Methods. (Values are means ± SD (mean of 6 mice/group); *P < 0.05.

Xenografted NOD/SCID mice treated with quercetin or green tea significantly decreased tumor volume compared with the control group. Treatment of mice with 120 mg/kg of quercetin resulted in 44% inhibition of tumor growth and with green tea treatment (100 mg/kg) resulted in 60% growth inhibition in HL-60 xenografts when compared to the control group at day 21 (Fig. 1A,B,C and D). As Fig. 1E and F showed, the body weights of the xenograft mice were not significantly variable between different treatment groups after 21 days.

### Quercetin and Green tea induce apoptosis of leukemic cells

**Figure 2:**
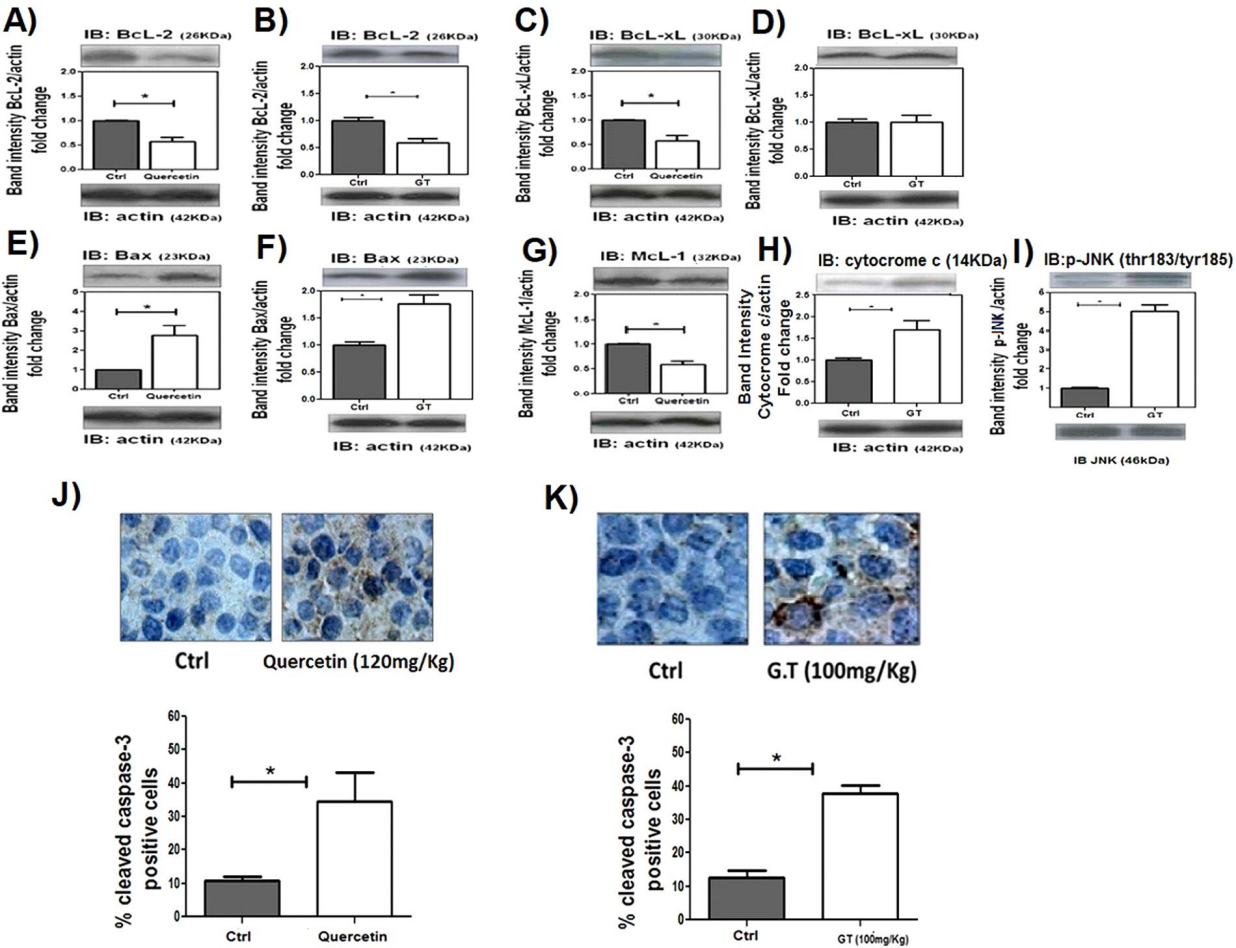
Quercetin and Green tea induces apoptosis of leukemic cells. NOD/SCID mice with HL60 tumors were treated or not with quercetin (120 mg/kg), once every 4 days or green tea (100 mg/kg), every day. After 3 weeks, tumors were harvested and relative levels of protein in tumor lysates were determined by Western blotting (**A**,**B**) BCL-2; (**C**,**D**) BCL-XL; (**E**,**F**) BAX; (**G)** -MCL-1; (**H) -**cytocrome c; (**I) -**p-JNK. (**J**,**K**) IHC analysis of cleaved caspase-3 in tumor sections and the percentage of caspase 3 positive nuclei of cells per field; four fields per tumor section. Counts were made using x40 objective connected to a light microscope (Olympus CBA; Olympus America). Values are means ± SD (mean of 6 mice/group); *P < 0.05.

The BCL-2 family contains a growing number of members that reduces cell death, including BCL-X, MCL-1. In this study, the expression of BCL-2 and BCL-XL antiapoptotic proteins was reduced by quercetin treatment (Fig. 2A,B) whereas expression of BAX, a proapoptotic protein, was increased. Therefore, as shown in Fig. 2G, a marked decrease of MCL-1 expression was observed in quercetin-treated HL-60 xenograft tumors as compared with the control group. Green tea treatment also decreased BCL-2 expression accompanied with noticeably increase in BAX, cytocrome c and p-JNK expression (Fig. 2), however no differences were observed in the expression of BCL-XL. Confirming the increased apoptosis of the cells, caspase-3 was activated to a greater extent after quercetin or green tea treatment (Fig. 2J–K). Cleaved caspase 3 staining was positive in 6.10% ± 0.73 cells in control group, whereas quercetin induced 41.73% ± 6.9 cells and the green tea treated group presented 45.68% ± 6.9 positive cells.

### Quercetin and green tea induce cell cycle arrest of leukemic cells

**Figure 3:**
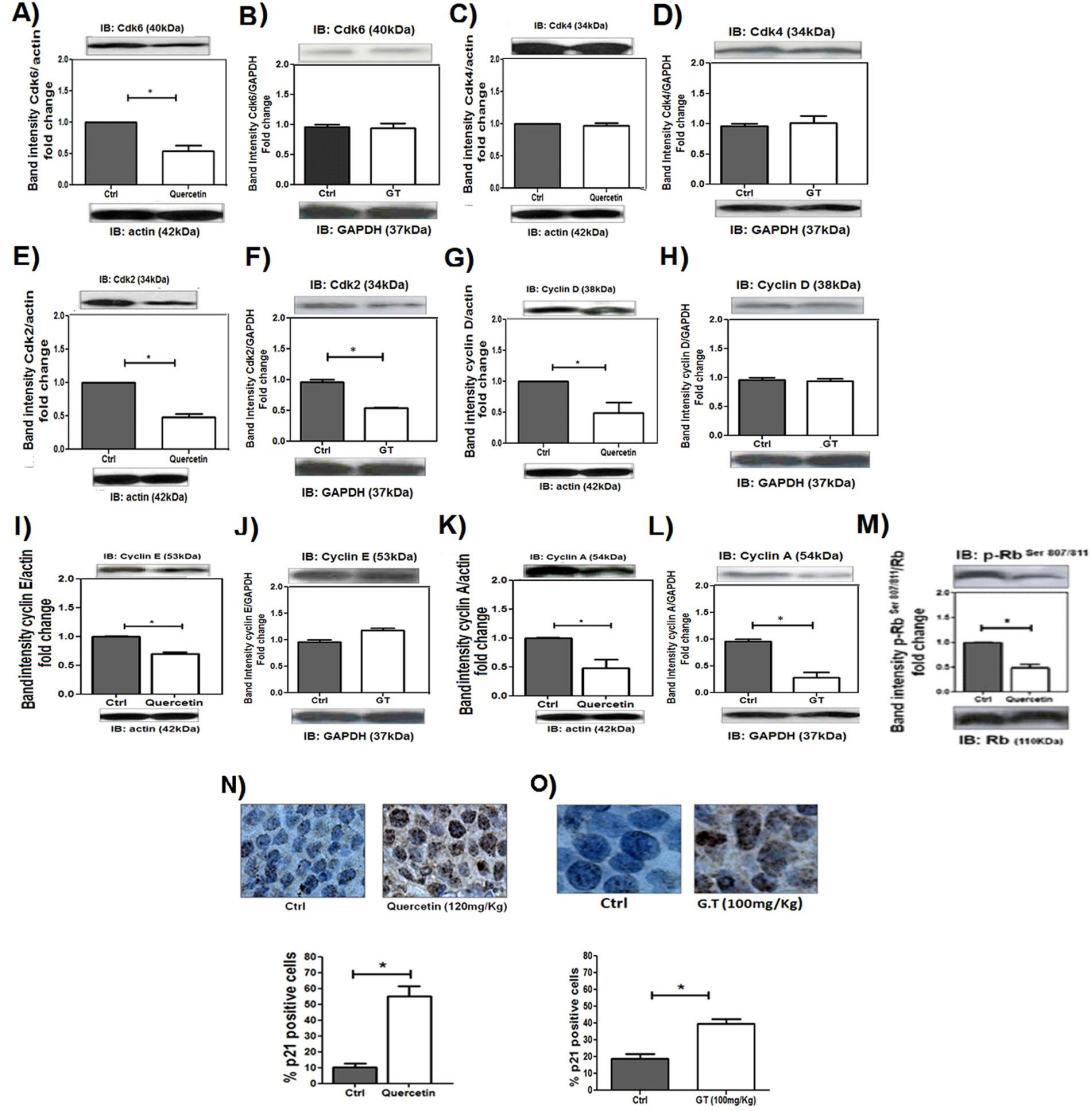
Quercetin and Green Tea induces arrest of the G1 phase of cell cycle in HL-60 xenografts. NOD/SCID mice with HL60 tumors received or not quercetin (120 mg/kg), once every 4 days or green tea (100 mg/kg), every day. After 3 weeks, tumors were harvested and relative levels of protein in the tumor lysates were determined by Western blotting; (**A**,**B**) CDK6; (**C**,**D**) CDK4; (**E**,**F**) CDK2; (**G**,**H**) CYCLIN D; (**I**,**J**) CYCLIN E; (**K**,**L**) CYCLIN A; (**M**) p-RB. (**N**,**O**) IHC analysis of p21 protein in tumor sections and the percentage of p21-positive nuclei of cells per field; four fields per tumor section. Counts were performed using x40 objective connected to a light microscope (Olympus CBA; Olympus America). Values are means ± SD (mean of 6 mice/ group); *P < 0.05.

To investigate the effects of quercetin and green tea in cell cycle arrest, expression of cell cycle regulatory proteins were examined. Western blot analysis showed that 3 weeks of quercetin treatment in xenograft model resulted in a pronounced decrease in protein levels of CDK6, CDK2, CYCLIN D, CYCLIN E and CYCLIN A (Fig. 3). The quercetin treatment also resulted in Rb-phosphorylation loss (Fig. 3M).

According to these results, green tea treatment induced decreased expression of CDK2 and CYCLIN A proteins (Fig. 3). Moreover, green tea and quercetin treatment resulted in pronounced p21induction (44% ± 5,6 and 54.54% ± 8.10 respectively) compared to controls (approximately 12% ± 2.61) (Fig. 3N,O). These results indicated that quercetin and green tea mediated G1 phase cell cycle arrest in HL-60 xenografts.

### Quercetin and green tea increase autophagy in leukemic cells

**Figure 4:**
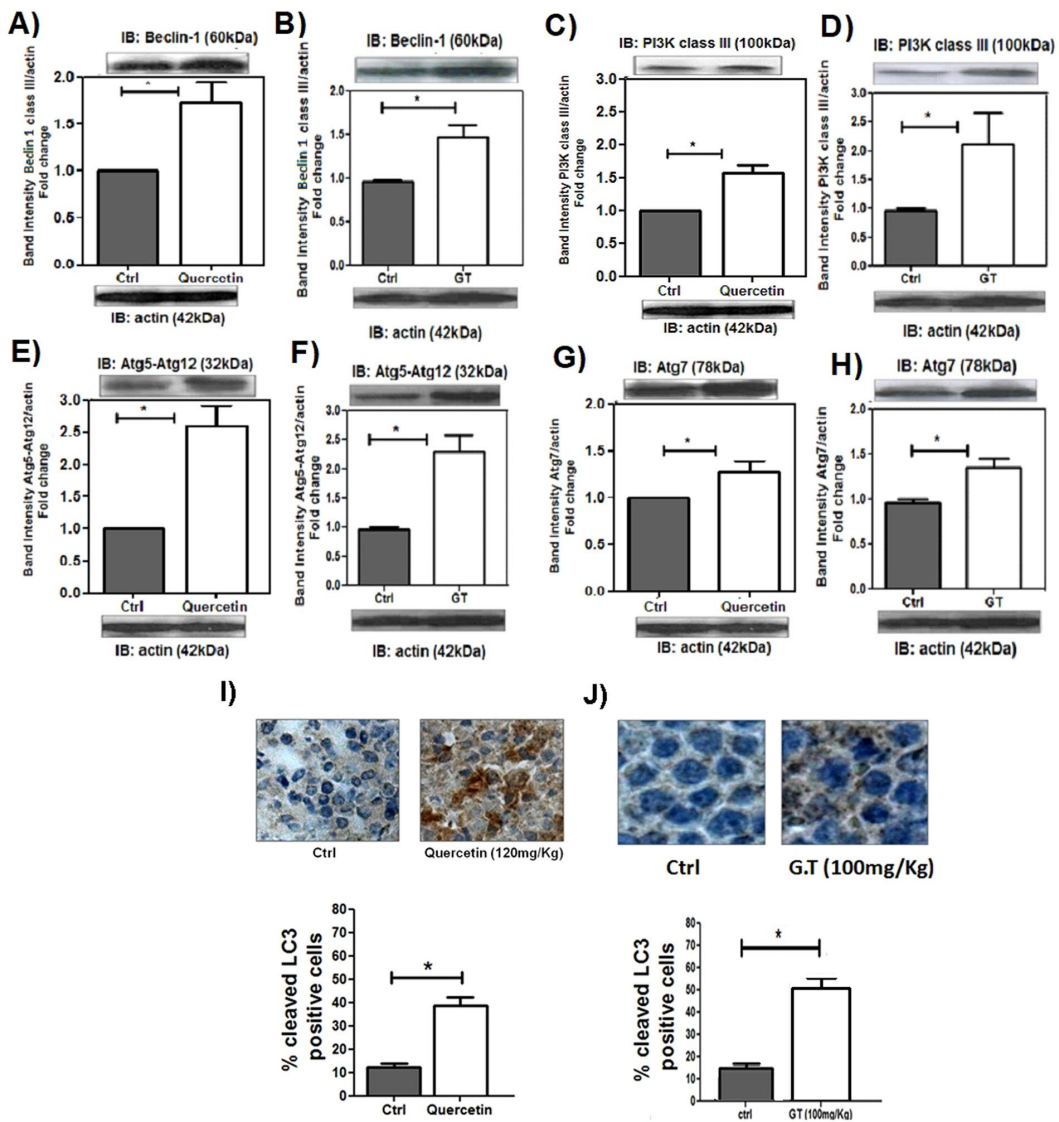
Quercetin and Green Tea induce autophagy in HL60 xenografts. NOD/SCID mice with HL60 tumors were treated or not with quercetin (120 mg/kg), once every 4 days or green tea (100 mg/kg), every day. After 3 weeks, tumors were harvested and relative levels of protein in tumor lysates were determined by Western blotting; (**A**,**B**) BECLIN-1; (**C**,**D**) PI3K; (**E**,**F**) ATG5-ATG12; (**G**,**H**) ATG7; (**I**,**J**) IHC analysis of cleaved LC3 in tumor sections and the percentage of LC3-positive nuclei of cells per field; four fields per tumor section. Counts were performed using x40 objective connected to a light microscope (Olympus CBA; Olympus America). Values are means ± SD (mean of 6 mice/group); *P < 0.05.

To examine whether autophagy markers were activated by quercetin or green tea, immunoblot analyses were utilized to measure expression of BECLIN 1, ATG7 and ATG12-ATG5. As a result, we found that both quercetin and green tea upregulated expression of all these autophagy-related proteins in HL-60 xenografts (Fig. 4). To further characterize this process, we assessed one of key hallmarks of autophagy: the conversion of LC3-I to LC-3-II. As shown in Fig. 4I,J, treatment with quercetin or green tea resulted in a pronounced induction of LC3-I to LC3-II switch.

## Discussion

Pharmacological exploitation of natural compounds has continued to lead to the development of non-synthetic and non-toxic anticancer agents that are promising for treatment of neoplasic diseases, such as leukemia^14^. This study aimed to determine the antileukemic activities of quercetin and green tea in HL-60 xenograft model.

It has been reported that administration of quercetin and green tea eliminate human breast cancer MDA-MB-435 cell xenografts as well as prostrate tumor xenografts^15,16^. In our *in vivo* studies, using HL-60 xenograft model, tumor volumes were reduced compared to controls after quercetin and green tea treatment, indicating an antileukemia activity of these compounds. To further validate the antileukemia activity, we next examined the apoptotic mechanism through the expression of pro and anti-apoptotic proteins. Increased BAX and decreased BCL-2 and MCL-1 expression were detected in the quercetin and green tea-treated xenografts compared with the control group. The MCL-1 was found to cause viability enhancement in a wide range of hematopoietic cell types, including lymphoid as well as myeloid cells at both immature and mature stages of differentiation^17^. The decrease in anti-apoptotic proteins was accompanied by release of cytocrome c in the green tea treatment. Once released to the cytoplasm, cytochrome c allows activation of caspases which results, in the last instance, in the activation of caspase 3. Our results confirm this cascade of events since we observed a significant increase in the expression of caspase 3 in animals inoculated with HL-60 and treated with quercetin or green tea.

Tumor development and progression have been shown to be dependent on cellular accumulation of various genetic and epigenetic events, including alterations in the cell-cycle machinery^18^. Previously, we have demonstrated that, in P39 xenografts, quercetin blocks cell-cycle progression at G1 phase and exerts this effect through p21 increase^7^. Another study showed, in U937 leukemic cells, the activity of green tea in the cell cycle by regulating the activity of G2/M phase proteins with the activation of p21^19^. Corroborating these results, we herein demonstrate an antiproliferative activity of quercetin and green tea. After treatment, the HL-60 cells inoculated in mice were retained in the G1 phase and we observed a decrease in CYCLIN A as well as CDK2 expression. In quercetin treatment, a significant alteration in CYCLIN D1, CYCLIN E, CDK 6 and in p-Rb activity was also observed. Moreover, p21 expression was significantly increased after quercetin and green tea treatment, which corroborates the finding of CYCLIN A and CDK2 decrease, since the presence of p21 prevents CYCLIN A-CDK2 complex formation. These are very important results as leukemic cells often become resistant to most drugs used in chemotherapy, through mechanisms that block the anti-proliferative and apoptotic effects thus, the development and/or discovery of agents that have the ability to modulate the cell cycle is promising.

In addition to the apoptosis and cell cycle, autophagy is a process capable of regulating the proliferative activities of cells. Autophagy is a bulk degradation process that promotes survival under metabolic stress, but it can also be a means of cell death if executed to completion^20,21^. During autophagy, a series of autophagy genes associated with autophagosome formation are activated^22^. In mammalian cells, PI3K can bind to BECLIN-1 to form the BECLIN-1/PI3K complex, and then participate in the mediation of phosphorylation and ubiquitination, eventually inducing autophagy. ATG7, a key autophagy protein, contributes to form autophagic vascuoles and activates ATG12. Attachment of ATG12 to ATG5, ATG12-ATG5 conjugate, localizes to autophagosome precursors^23^. Defects in autophagy by loss of BECLIN-1 function or deficiency in ATG5 have been reported to promote gene amplification and chromosomal instability, suggesting that autophagy plays an important role in the maintenance of genomic integrity^24^. Our results show the ability of quercetin and green tea to stimulate autophagic activity in HL-60 xenografts. Quercetin and green tea were able to modulate this activity by increasing BECLIN-1, PI3K, ATG5-ATG12, ATG7 and cleavage of LC3I in LC3II, corroborating studies that showed quercetin^7^ and green tea^25^ ability to modulate the autophagic process positively.

BECLIN-1 is constitutively inhibited through BCL2 and BCL-XL interaction. JNK signaling can weaken this interaction and activate BECLIN-1. Shimizu *et al*.^26,27^ confirmed the crucial role of JNK activation in the autophagic cell death of embryonic fibroblasts from BAX/BAK double-knockout (DKO) mice. While survival-dependent autophagy does not require JNK activation, this pathway may be fundamental to promote autophagy cell death. Our results corroborate this hypothesis, since green tea was able to activate the JNK signaling pathway as well as BECLIN-1, LC3II increase and decrease of BCL2 expression in HL-60 xenografts.

Finally, we conclude that the use of polyphenolic compounds may be an adjuvant in the treatment for leukemia, as the unbalance of cell death and proliferation processes is important in the pathogenesis of leukemia. Moreover, the use of *in vivo* tumor models is a reliable tool for translation medicine and the results here obtained should be explored in clinical trials.

## Materials and Methods

### Reagents and antibodies

Quercetin (>98% pure), and Green tea were obtained from Sigma. The CYCLIN D, cyclin E, CYCLIN A, CDK4, CDK2, CDK6, P21, P27, BCL-XL, BAX, BCL-2, cytocrome c, MCL-1, actin, and GAPDH from Santa Cruz Biotechnology. BECLIN-1 and PI3K class III from Cell Signaling Technology. pJNK from Invitrogen. ATG5-ATG12, ATG7 and LC3I/II from Abcam Inc. Anti-rabbit, anti-mouse, and anti-goat peroxidase–conjugated antibodies from KPL, Inc. The HL-60 cell line, derived from a 36-year-old woman with acute promyelocytic leukemia, was obtained from ATCC, Philadelphia, PA. Cytogenetic analyses of the cell line, during the procedures of this study, identified the same alterations as previously described^27^ in 100% of metaphases analysed.

### Human tumor xenograft model

Female (NOD.CB17-Prkdcscid/J lineage) 6- to 8-week-old animals, from The Jackson Laboratory, bred at the Animal Facility Centre at the University of Campinas, under specific pathogen-free conditions, were matched for bodyweight before use. the Experimental protocol was approved by CEUA, an Institutional Committee of Animal Care. Mice were inoculated, s.c., in the dorsal region, on day 0 with 0.1 mL of HL-60 (1 × 10^7^ cells/mice). Every 7 days tumor volumes were evaluated according to the formula: tumor volume (mm^3^) ¼ (length × width2)/2. Green tea or Quercetin treatment was initiated after tumors reached 100 to 200 mm^3^. Quercetin was administered once every 4 days by i.p injection at 120 mg/kg body weight and Green tea every day by gavage at 100 mg/kg body weight. The control group received equal amounts of vehicle solution, as previously described^17^. Mice were sacrificed after 21 days, tumors were then removed, minced, and homogenized in protein extraction buffer or immediately fixed in formalin for IHC.

### Western blot analysis

Total cell protein was extracted in RIPA buffer. Protein concentrations were quantified by the Bio-Rad Protein Assay Kit. Equal protein amounts were loaded on 8% to 15% SDS polyacrylamide gels and electrophoretically transferred to nitrocellulose membrane. Nonspecific binding sites were blocked by incubation with a buffer containing Tris (10 mmol/L, pH 7.4), NaCl (150 mmol/L), Tween 20 (0.1%), and fat-free dry Milk (5%). Membranes were incubated overnight with a specific primary antibody, at 4 °C, followed by horseradish peroxidase–conjugated secondary antibody, at room temperature for 1 hour. Immunoreactivities were visualized by ECL Western Blot Analysis System (Amersham Pharmacia Biotech).

### Immunohistochemistry

Active caspase-3, p21 and LC3I/II expression were measured on paraffin-embedded sections using conventional immunohistochemical techniques. Briefly, a 4 mmol/L tumor section was dewaxed and rehydrated. Antigen retrieval was performed by pretreatment of slides in citrate buffer (pH 6.0) in a microwave oven for 12 minutes, sections were then incubated overnight with monoclonal antibodies of interest at 4 °C. The reaction was detected with the streptavidin–biotin–peroxidase complex and stained with diaminobenzidine. Counterstaining was performed with Meyer’s hematoxylin.

### Statistical analysis

For comparison, an appropriate Student t test or ANOVA was performed. All statistical analyses were carried out using Prism version 5.0a software (GraphPad Software) and results are considered significant when P < 0.05.
